# SG-APSIC1129: Long-term effect of a bundled care program in reducing central-line–associated bloodstream infections

**DOI:** 10.1017/ash.2023.54

**Published:** 2023-03-16

**Authors:** Yingchieh Liu, Ying-Chieh Liu, Kuan-Yin Lin, Chi-Tai Fang, Yu-Jing Chang, Sung-Ching Pan, Jen-Tay Wang, Wang-Huei Sheng, Yee-Chun Chen, Jia-Horng Kao, Shan-Chwen Chang

**Affiliations:** National Taiwan University Hospital, Taiwan; Taiwan, National Taiwan University Hospital, Taipei, Taiwan; National Taiwan University Hospital, Taipei, Taiwan; National Taiwan University Hospital, Taipei, Taiwan; National Taiwan University Hospital, Taipei, Taiwan; National Taiwan University Hospital, Taipei, Taiwan; National Taiwan University Hospital, Taipei, Taiwan; National Taiwan University Hospital, Taipei, Taiwan; National Taiwan University Hospital, Taipei, Taiwan; National Taiwan University Hospital, Taipei, Taiwan; National Taiwan University Hospital, Taipei, Taiwan

## Abstract

**Objectives:** Central-line–associated bloodstream infection (CLABSI) has been the leading cause of healthcare-associated infections (HAIs) in the intensive care unit (ICU) setting. Previous studies have shown that a care bundle is effective in reducing CLABSI rates; however, the data on long-term sustainability and cost savings of bundled care are limited. **Methods:** From January 2011 to December 2020, a prospective surveillance was performed to monitor CLABSI at a university hospital in northern Taiwan. To reduce the CLABSI rate, a hospital-wide bundled care program for CLABSI prevention was implemented in 2013. We evaluated the long-term effect of the care bundle on CLABSI incidence and length of stay in the ICU. **Results:** During the study period, the overall CLABSI incidence decreased from 8.22 per 1,000 catheter days before the care bundle was implemented to 6.33 per 1,000 catheter days in 2020 (*P* for trend <.01). The most common pathogens causing CLABSI were gut organisms (1,420 of 2,363, 60.1%), followed by environmental organisms (734 of 2,363, 31.1%) and skin organisms (177 of 2,363, 7.5%). The decreasing trend was statistically significant in the incidence of CLABSI caused by skin organisms (*P* for trend < .01), but not in the incidence of CLABSI caused by environmental organisms (*P* for trend = .86) or gut organisms (*P* for trend = .06). In the multivariable analysis, implementation of this care bundle was independently associated with a decrease in the CLABSI rate (RR, 0.77; 95% CI, 0.66–0.88). Compared with patients without CLABSI, patients with CLABSI had a longer average ICU length of stay (27 vs 17 days). **Conclusions:** A sustainable reduction in the incidence of CLABSI caused by common commensals could be achieved through a cost-saving bundled care program.

